# Exposure to tobacco imagery in streaming television is associated with increased intentions to smoke and vape

**DOI:** 10.1016/j.abrep.2026.100668

**Published:** 2026-01-13

**Authors:** Nathan A. Silver, Brenda Dimaya, Elexis C. Kierstead, Madison Iskra, Maeh Al-Shawaf, Michael A. Tynan, Jessica M. Rath

**Affiliations:** a Truth Initiative Schroeder Institute, Washington, DC, United States; b Department of Health, Behavior and Society, Johns Hopkins Bloomberg School of Public Health, Baltimore, MD, United States; c Department of Behavioral and Community of Health, University of Maryland School of Public Health, College Park, MD, United States; d Unaffiliated

**Keywords:** Television, Entertainment media, Smoking, Vaping, Tobacco

## Abstract

**Introduction:**

Streaming platforms have changed the way we watch television. Although previous research has established a causal relationship between tobacco exposure on-screen and tobacco use, it’s unclear whether changes in the media environment brought about by streaming have altered this process. We hypothesize that youth and young adult’s intentions to use cigarettes and e-cigarettes in the future will be positively associated with greater exposure to tobacco imagery in streaming television.

**Methods:**

A content analysis identifying the amount of tobacco imagery in 60 popular streaming shows with a new season airing between 2019–2022 and an online survey of 1032 15–24-year-olds were conducted to examine the relationship between exposure to tobacco imagery in streaming television and intentions to use.

**Results:**

Compared to those with low exposure to tobacco imagery, moderate and high exposure was associated with 61% and 176% respectively higher odds of increased intentions to use e-cigarettes, and 104% and 168% respectively higher odds of increased intentions to smoke cigarettes. Significant interactions indicate that the relationship between high exposure and intentions to use e-cigarettes and cigarettes was weaker among those who had used a tobacco or nicotine product in the past 30 days.

**Conclusion:**

The relationship between exposure to tobacco imagery and intentions to use tobacco or nicotine products in the future endures on streaming media. Future research is needed to parse differential influences on initiation versus cessation and better understand what kinds of depictions drive these relationships.

## Highlights

• Median exposure to tobacco imagery in streaming television was 2066 incidences.
• A dose response relationship was found between exposure and behavioral intentions.
• Tobacco imagery may have a different impact on initiation versus cessation.

## 1 Introduction

The prevalence and influence of tobacco imagery in entertainment media has long been linked to tobacco use in young people (Dal Cin et al., 2012, Gutschoven and Van den Bulck, 2005, Nunez-Smith et al., 2010). Citing this research in 2012, the U.S. Surgeon General concluded that there is a causal relationship between exposure to depictions of smoking in movies and smoking initiation among young people (Surgeon General, 2012). Subsequent research turned towards television, identifying tobacco imagery in 86% of broadcast and streaming shows most popular among young people between 2015 and 2017 (Rath et al., 2020). A longitudinal examination then showed that exposure to such programming predicted tripled odds of initiating e-cigarette use (Bennett et al., 2020) signaling that exposure to tobacco in entertainment media likely causes use in real-life.

The rise of on-demand streaming sites such as Netflix have changed the way people, specifically youth and young adults, watch television. Streaming enables more personalized media diets with showrunners even adapting characteristics of shows to capitalize on the ability to watch multiple episodes in a row (Flayelle et al., 2020, Netflix, 2013, Onyeaka et al., 2024). Despite the wider availability of diverse content, social motivations like fear of missing out were more often reported as the reason for streaming-specific behaviors like binge-watching (Anghelcev et al., 2022). Similarly, motivations like relaxation and escapism, while not unique to streaming content, may drive content exposure to a greater extent on streaming compared to broadcast (Leiner and Neuendorf, 2022, Tefertiller and Sheehan, 2019). Content analyses of popular streaming television (Truth Initiative, 2023) as well as shows and movies developed specifically for streaming platforms (Allem et al., 2022) show that depictions of tobacco use remain prevalent. Given the shift from broadcast to streaming in the past decade (Nielsen, 2025), it is important to reexamine the relationship between exposure to tobacco use and intentions to use in real life amongst a generation whose television consumption takes place predominantly on these streaming platforms.

The current study examines exposure to tobacco use imagery among U.S. 15–24 year olds on streaming platforms, the dominant mode of television viewing in the U.S. (Park & McClain, 2025). We ask how often are youth and young adults exposed to tobacco imagery in the streaming programming they watch (RQ1)? We then hypothesize that like broadcast, greater exposure to depictions of tobacco imagery in streaming media will be associated with greater intentions to smoke cigarettes (H1) and to use e-cigarettes (H2) in the future. Finally, given consistent findings that television has a stronger influence when real-life experience is low (Schnauber & Meltzer, 2015), we expect the relationship between exposure and intentions to be moderated by previous tobacco use (H3).

## 2 Methods

### 2.1 Procedure

**Fig. 1:**
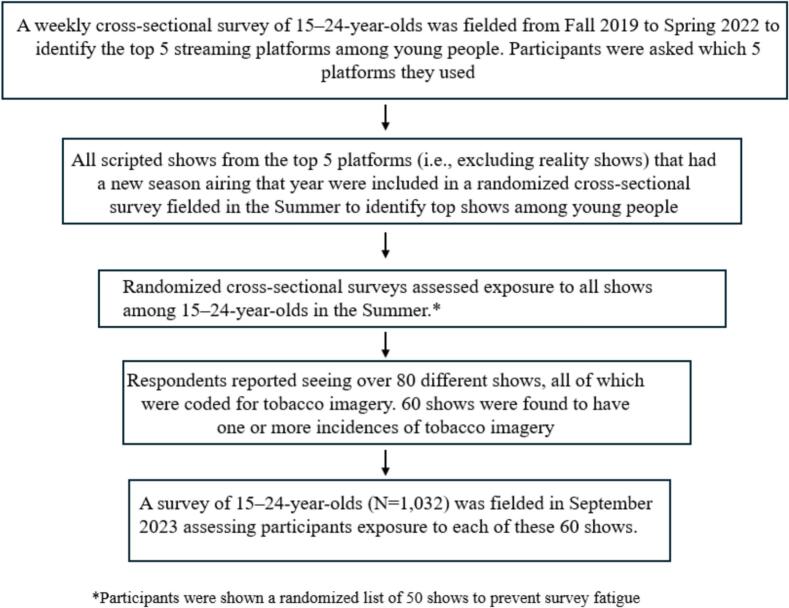
Outline of the methodology used to identify popular shows and streaming platforms from Fall 2019 to Spring 2022.

A weekly tracking survey of U.S. 15–24-year-olds was used to identify popular scripted shows (i.e. excluding reality shows) with new seasons airing between 2019–2022 across streaming platforms (see timeline in Fig. 1). All shows were content analyzed using the peer-reviewed *Thumbs Up Thumbs Down* methodology to quantify tobacco imagery depictions (Glantz et al., 2010, Rath et al., 2020, Tynan et al., 2017). Two independent coders watched each episode in full, recording timestamps of tobacco or nicotine depictions including both use as well as imagery like scenes taking place in retail environments selling tobacco or nicotine products. All discrepancies were adjudicated via discussion with a third rater. Descriptive results of this study are published in an annual report (Truth Initiative, 2025). Estimated per-episode exposure to tobacco imagery was then calculated for all shows. An online survey of U.S. 15–24-year-olds fielded between September 21–25 of 2023 assessed self-reported exposure in the past year to each of 60 shows containing tobacco imagery along with demographic covariates, past 30-day tobacco use, and future intentions to smoke cigarettes and vape e-cigarettes. All study protocols were reviewed and approved for human participant research by Advarra Institutional Review Board (Pro00033267).

### 2.2 Sample

Qualtrics fielded an online survey of N = 1,032 15–24-year-olds. Quotas were set for race/ethnicity to power potential analyses related to racial and ethnic disparities and to oversample (at least 30%) for past 30-day tobacco use (Table 1).

**Table 1:** Sample characteristics among youth and young adults aged 15–24-year-olds (n = 1032).

	*n*	(%)
Female	525	(50.87%)
Hispanic	263	(25.48%)
Black	255	(24.71%)
Asian	46	(4.46%)
Rural residence	267	(25.87%)
Past 30-day tobacco use	328	(31.78%)
	***M***	**(*SD*)**
Age	20.9	(0.1)

### 2.3 Measures

#### 2.3.1 Estimated tobacco exposure dosage

For each of the 60 television shows dosage was calculated in two steps. First, participants estimated how much of each series they had watched in the last year on a four-point scale from “none” to “watched all episodes”. In step two, the ratio of total incidents of tobacco imagery per episode in the series was calculated. For example, a 10-episode series with a total of 100 coded incidences of tobacco imagery could result in a dosage of 0 (none), 10 (one episode or less), 50 (watched multiple episodes), or 100 (watched all episodes). Each participants’ dosage represents an aggregate estimate of the number of times they were exposed to tobacco imagery while watching 1–60 of the shows in the last year.

#### 2.3.2 Demographics

We controlled for potential confounders related to known demographic correlates of tobacco use. Participants reported their age, sex, race/ethnicity, and rurality. Dummy codes were created for female, Black/African American, Hispanic, Asian and rural (residing in a small town or rural area) participants with the absence of these characteristics as the reference group.

#### 2.3.3 Tobacco Use and intentions

Participants indicated whether they had used cigarettes, e-cigarettes, cigars, little cigars or cigarillos, hookah, pipe tobacco, loose tobacco, and oral nicotine products in the past 30 days. This variable was dummy coded to differentiate past 30-day users from all other participants. The implications of the heterogenous reference group are discussed in the limitations below. Finally, intentions to “use an e-cigarette” and “smoke a cigarette” in the next year were assessed as our primary dependent variables. Participants responded on a four-point scale that they would “definitely not”, “probably not”, “probably yes”, or “definitely yes”.

### 2.4 Analysis

Estimated exposure dosage ranged from 0 to 7,489.5; Mdn[IQR] = 2066. The median and IQR were used to group viewers such that *low exposure* fell beneath the IQR (dosage < 1168), *moderate exposure* fell within the IQR (dosage between 1,168.1–3,075), and *high exposure* was above the IQR (dosage > 3075). *Low exposure* was used as the comparison group for all regression analyses. We fit Ordinal Logistic Regression models to examine the relationship between dosage and intentions to use e-cigarettes and smoke cigarettes. Odds ratios were adjusted for age, sex, race/ethnicity, rurality, and the interaction between exposure and past 30-day tobacco use. Brant tests were used to test the proportional odds assumption (Liu et al., 2023).

## 3 Results

Table 2 provides odds ratios and 95% confidence intervals for ordinal logistic regression analyses predicting intentions to use e-cigarettes and smoke cigarettes. Female participants had 59% higher odds of increased intentions to use e-cigarettes and 39% higher odds of increased intentions to smoke cigarettes. Holding other variables constant, participants who used tobacco products in the past-30-days were 25x more likely to report increased intentions to use e-cigarettes and nearly 9.5x more likely to report increased intentions to smoke. A robust dose–response relationship was observed for both e-cigarette use and smoking intentions. Compared to those with low dosage, moderate dosage was associated with 63% higher odds of increased e-cigarette use intentions and 104% higher odds of increased smoking intentions while high dosage was associated with 176% higher odds of increased e-cigarette use intentions and 168% higher odds of cigarette use intentions. Significant interactions between past 30-day tobacco use and high but not moderate dosage suggest the observed relationship between high dose and intentions is 57% weaker for e-cigarette use intentions and 59% weaker for smoking intentions among participants who used tobacco products in the past 30-days compared to those who have not. The Brant test was not significant for the e-cigarette intentions model (p = 0.144) but was significant for the smoking intentions model (p = 0.018). This violation of the proportional odds assumption was driven by rurality (p = 0.032), which itself was not a significant predictor in the model. Nevertheless, we interpret the odds ratio for rurality with caution considering this violated assumption.

**Table 2:** Ordinal logistic regression predicting behavioral intentions (N = 1032).

	E-Cigarette Use Intentions		Smoking Intentions	
	OR	95% CI	OR	95% CI
Age	0.99	0.94–1.05	1.02	0.97–1.08
Female	1.6	1.20–2.12***	1.39	1.03–1.86*
Black	0.75	0.54–1.02	0.79	0.57–1.10
Hispanic	0.91	0.67–1.24	0.97	0.71–1.33
Asian	0.87	0.44–1.72	1.60	0.84–3.04
Rural	1.14	0.85–1.53	1.19	0.88–1.62
Past 30-day tobacco use	26.05	13.27–51.15***	10.53	5.23–21.20***
Moderate dosage^a^	1.61	1.06–2.45*	2.04	1.30–3.19**
High dosage^b^	2.76	1.67–4.55***	2.68	1.57–4.58***
Moderate dosage x past 30-day tobacco use^c^	0.57	0.27–1.20	0.58	0.26–1.26
High dosage x past 30-day tobacco use^c^	0.43	0.19–0.96*	0.41	0.17–0.95*
Model fit Χ^2^(df)	448.57(11)***	229.25(11)***		

* p < 0.05, **p < 0.01, ***p < 0.001; ^ab^base category is low exposure to tobacco imagery while streaming (the bottom 25th percentile). Moderate exposure is comprised of the middle 50% (26th through 74th percentiles), while high exposure is comprised of the top 25th percentile. ^c^the interaction terms between dosage and past 30-day tobacco use tests the significance of past 30-day tobacco use as a moderator of the relationship between exposure to tobacco content while streaming and tobacco use intentions.

## 4 Discussion

This study has two main findings with implications for the impact of entertainment media on tobacco use. First, more than half of our sample of U.S. youth and young adults were exposed to 2000 or more tobacco depictions through the shows they watched in 2023. Moreover, this estimate is conservative as it only accounts for shows with new seasons airing between 2019 and 2022. Second, a robust dose–response relationship between the amount of exposure to tobacco imagery and intentions to use cigarettes and e-cigarettes adds to the body of literature highlighting the relationship between prevalent depictions of tobacco and nicotine products on T.V. and real-world tobacco use among youth and young adults. This relationship is significantly stronger for participants who had not used tobacco products in the past 30 days indicating the need for further research aimed at disentangling the influence of television on tobacco or nicotine initiation, escalation, and cessation-related outcomes.

Our findings corroborate previous research on the prevalence and influence of tobacco imagery in entertainment media (Allem et al., 2022, Bennett et al., 2020, Rath et al., 2020; Surgeon General, 2012), showing that despite changes to the tobacco media environment the normalization of tobacco use on popular television remains a dominant theme. Importantly, our baseline category was not “no exposure to tobacco content,” but “low exposure,” accounting for more than 1000 incidents of tobacco use on screen in the past year among the 25% of respondents with the lowest estimated dosage. Participants with a more typical dosage such as the median of over 2000 incidents, or the top 25% of respondents with over 3000 incidents had from 50% to over 150% increased odds of reporting greater intentions to use cigarettes and e-cigarettes. Combined with causal evidence of the effect of exposure to televised depictions of tobacco use from previous studies (Bennett et al., 2020), the robust dose–response association between exposure and intentions in our study, while not causal itself, highlights the risk posed by a seemingly ubiquitous characteristic of streaming television. Such findings underscore the need for proven intervention strategies to combat problematic media influences such as media literacy training (Donaldson & Allem, 2023).

The significant interaction between exposure and past 30-day use is suggestive of ceiling effects, as irrespective of exposure to tobacco imagery in media, current users undoubtedly have higher intentions to keep using than non-users. Moreover, research on the influence of television shows that effects are typically moderated by personal experience (Schnauber & Meltzer, 2015). Thus, while never users acquire tobacco supportive beliefs, norms, and attitudes that may lead to initiation from media depicting tobacco use (Gutschoven & Van den Bulck, 2005), those who have experience with tobacco outside media are likely less influenced by televised depictions.

This interplay between personal experience and “mediated” experience through television provides the most fertile ground for future research. In general, television’s ability to simulate vicarious experience (Bandura, 2001, Mar and Oatley, 2008) is a key driver through which exposure influences health relevant outcomes (Bandura, 2004, Braddock and Dillard, 2016, Moyer-Gusé, 2008, Slater and Rouner, 2002). Future research aiming to understand whether the 21st century media environment alters the effect of tobacco’s ubiquitous depiction in television should examine how such changes influence the content viewers see and the subjective viewer experience. Although the exposure to behavior pathway is strongly supported, the extent to which entertainment media can facilitate influential vicarious experiences is ever evolving.

### 4.1 Limitations

This study has several limitations. This study alone does not allow for causal inference. Moreover, generalization of effect sizes and population parameters should be interpreted with caution given the use of a convenience sample. Second, our exposure dosage estimation is blunt and treats all imagery as equal. The granularity of the dosage measure allowed for no exposure, one episode, half a series, or all of a series. While show viewership varies at other intervals, we believe these intervals effectively balanced limitations related to survey fatigue and recall for participants asked to recall exposure to 60 TV shows. The presence of tobacco content within these shows is measured objectively, allowing for strong estimates based on self-reported exposure to the shows rather than recall of tobacco imagery. However, we also note that the nature of the content coding process (with a third adjudicator for all content) did not allow for traditional interrater reliability metrics to be calculated. Third, our use of past 30-day use as our current use measure introduces error to the distinction between initiation, escalation, and cessation-related behaviors. Intentions for a non-past 30-day user are likely indicative of potential initiation but could also indicate potential relapses or even escalation for an experimental user. Future research should aim to disentangle television’s effect on these psychologically distinct processes. Finally, the violation of the proportional odds assumption for rurality is most likely a symptom of a blunt binary measurement of a complex geographical construct. Thus, interpreting the relationship between rurality and tobacco use based on these data is inappropriate.

## 5 Conclusion

Tobacco use remains highly prevalent across the television programs youth and young adults watch. Meanwhile the media environment continues to evolve in ways that may attenuate, amplify, or otherwise alter the influence of entertainment media on health behaviors like tobacco use. Research examining key processes that drive television’s influence can inform both strategies to mitigate harmful effects of on-screen tobacco imagery, as well as media-based interventions for prevention and cessation.

## Declaration of Generative AI and AI-assisted technologies in the writing process

No AI or AI-assisted technologies were used in the writing process.

## CRediT authorship contribution statement

**Nathan A. Silver:** Writing – review & editing, Writing – original draft, Visualization, Methodology, Investigation, Formal analysis. **Brenda Dimaya:** Writing – review & editing, Writing – original draft. **Elexis C. Kierstead:** Writing – review & editing, Writing – original draft. **Madison Iskra:** Visualization, Software, Formal analysis. **Maeh Al-Shawaf:** Writing – review & editing, Writing – original draft. **Michael A. Tynan:** Writing – review & editing, Writing – original draft. **Jessica M. Rath:** Writing – review & editing, Writing – original draft, Supervision, Conceptualization.

## Funding

This study was funded internally by Truth Initiative. This research did not receive any specific grant from funding agencies in the public, commercial, or not-for-profit sectors.

## Declaration of competing interest

The authors declare that they have no known competing financial interests or personal relationships that could have appeared to influence the work reported in this paper.

## Data Availability

A data sharing agreement is required for use of all data. Truth Initiative does not share data with tobacco industry representatives or affiliated researchers. Investigators seeking access to data used in the study should make a written request to Truth Initiative authors and submit a detailed research plan including the purpose of the proposed research, required variables, duration of the analysis phase, IRB approval with FWA information and documentation of investigator training in human subjects. Approved investigators may access datasets via an analytic Portal owned and administered by Truth Initiative.
